# Effects of Gastric Bypass on Beta-cell function and Insulin sensitivity in Patients with Non-Severe Obesity (BMI<35 kg/m^2^) and Insulin-treated Type 2 Diabetes

**DOI:** 10.1007/s11695-026-08702-5

**Published:** 2026-04-27

**Authors:** Adisa Poljo, Annika Rühle, Stefan Kopf, Christine Mass, Peter P. Nawroth, Marko Kraljević, Jennifer M. Klasen, Romano Schneider, Ralph Peterli, Beat P. Müller, Amalia Gastaldelli, Julia Szendrödi, Adrian T. Billeter

**Affiliations:** 1 Department of Visceral Surgery, Clarunis, University Digestive Health Care Center Basel, St. Claraspital and University Hospital of Basel, Basel, Switzerland; 2 Department of General and Visceral Surgery, Klinikum Aschaffenburg, A-Burg, Germany; 3 Department of Internal Medicine I and Clinical Chemistry, University Hospital Heidelberg, Heidelberg, Germany; 4 German Center for Diabetes Research, Neuherberg, Germany; 5 Heidelberg University, Heidelberg, Germany; 6 Department of Clinical Research, University of Basel, Basel, Switzerland; 7 Cardiometabolic Risk Unit, Clinical Physiology Institute, Pisa, Italy; 8 Chirurgie Zentrum St. Anna, Hirslanden Hospital, Lucerne, Switzerland; 9 Department of Visceral Surgery, University Hospital of Basel, Basel, Switzerland

**Keywords:** Diabetes, T2D, Beta-cell function, Insulin resistance, Insulin sensitivity, Gastric bypass, RYGB

## Abstract

**Background:**

Roux-en-Y gastric bypass (RYGB) is an effective treatment for type 2 diabetes (T2D), but the physiological mechanisms underlying sustained improvements in glycemic control remain incompletely understood.

**Objective:**

To descriptively compare short- and long-term changes in glycemic control and insulin secretion dynamics following RYGB or best medical care (BMC) using an intravenous glucagon stimulation test (GST) in patients with non-severe obesity (BMI <35 kg/m^2^) and insulin-treated T2D.

**Methods:**

Thirty-five patients with long-standing insulin-dependent T2D were enrolled from two prospective studies and received either BMC or RYGB. GST was performed at baseline and repeated after 3, 6, 12 and 24 months. Changes in glucose, insulin, and C-peptide responses were analyzed. Exploratory indices derived from the GST were used to assess longitudinal within-subject changes.

**Results:**

Diabetes remission occurred in 10% of patients in the RYGB group versus 0% in the BMC group. Insulin therapy was discontinued in 73% versus 33%, while oral antidiabetic medication was discontinued in 33% vs. 0%. After 24 months HbA1c, BMI and use of antidiabetic medication were lower in the RYGB group. Insulin secretion rapidly decreased and remained lower over 24 months after RYGB, while C-peptide responses were largely stable and glycemic control improved. Exploratory GST-derived indices increased after RYGB but not after BMC.

**Conclusion:**

In this descriptive and exploratory analysis, RYGB was associated with changes consistent with improved metabolic efficiency, reflected by enhanced glycemic control and reduced insulin secretion requirements under intravenous stimulation testing conditions. These findings are hypothesis-generating and warrant confirmation in larger, methodologically controlled studies.

**Supplementary Information:**

The online version contains supplementary material available at https://doi.org/10.1007/s11695-026-08702-5.

## Highlights

RYGB reduced HbA1c and BMI over 24 months compared with BMC.

73% of patients undergoing RYGB discontinued insulin therapy.

Insulin AUC during GST decreased rapidly and remained reduced after RYGB.

C-peptide responses during GST were largely preserved.

**Supplementary Information:**

The online version contains supplementary material available at https://doi.org/10.1007/s11695-026-08702-5.

## Introduction

Metabolic bariatric surgery (MBS) is an effective treatment option for type 2 diabetes mellitus (T2D). Several randomized controlled trials and meta-analyses have shown that MBS achieves superior glycemic control and a much higher likelihood of T2D remission compared to lifefstyle interventions [1–4]. Furthermore, it has been demonstrated that long-term consequences of T2D such as micro- and macrovascular complications can be significantly reduced [5, 6]. Despite this clinical evidence, the underlying mechanisms of how MBS exerts these effects are still not fully understood [7–9].

Postoperative increases in gastrointestinal hormones, particularly glucagon-like peptide-1 (GLP-1), are thought to contribute to improved glycemic control independent of weight loss [10–12].

The incretin effect describes the stronger insulin secretion after oral glucose intake compared to intravenous glucose administration. Since GLP-1 is produced mainly in the ileum, the anatomical changes after Roux-en-Y gastric bypass (RYGB) lead to an enhanced GLP-1 response because nutrients and glucose bypass the stomach, duodenum and parts of the jejunum, reaching more distal segments of the intestine [13]. The small intestine length may also influence GLP-1 secretion, as longer segments provide more surface area for nutrient absorption and enteroendocrine stimulation [14]. In T2D, GLP-1 responses to oral glucose are blunted but restored after RYGB. While these effects of RYGB have been shown by several groups, most studies used an oral glucose or mixed-meal test to assess the effects of MBS [15, 16]. These approaches inherently stimulate gastrointestinal hormone secretion and are influenced by altered gastrointestinal anatomy after surgery, making it difficult to distinguish enteral from intravenous mechanisms.

Intravenous stimulation testing paradigms avoid acute enteral nutrient exposure and allow standardized assessment of insulin and glucose responses. Studies using euglycemic clamps or intravenous glucose tests have examined RYGB effects [17–19], but follow-up was limited to weeks, leaving long-term changes in glycemic control under intravenous testing conditions unknown.

This study investigated short- and long-term changes in glycemic control and insulin secretion dynamics following RYGB compared with best medical care (BMC) using GST [20] in patients with non-severe obesity with a body mass index (BMI) < 35 kg/m^2^ and insulin-treated T2D, with follow-up extending to 24 months.

## Materials and Methods

Patients were drawn from two prospective studies, DiaSurg 1 and 2. DiaSurg 1 was a single-arm cohort examining RYGB in patients with non-severe obesity (BMI < 35 kg/m²) and insulin-treated T2D (DRKS00004605), with some results previously published [21–25]. DiaSurg 2 was a multicenter RCT comparing RYGB with insulin-based medical therapy in similar patients who also had at least one microvascular complication (DRKS00004550) [26]. Inclusion criteria were insulin-treated T2D ≥ 3 months, BMI 25–35 kg/m², preserved insulin secretion (glucagon-stimulated C-peptide > 1.5 ng/ml), HbA1c > 7%, and age 18–70 years. DiaSurg 2 additionally required at least one microvascular complication such as a diabetic neuropathy, nephropathy or retinopathy. Exclusion criteria included diabetes not treated with insulin, impaired pancreatic function, severe organ dysfunction, or malignancy within five years. All procedures conformed to the 1964 Helsinki declaration and its later amendments or comparable ethical standards and were approved by the Ethics Committee of the Medical Faculty of the University of Heidelberg (reference numbers: S-078/2010 and S-285/2012).

Surgical patients of the DiaSurg 1 study and the patients randomized to the surgical arm in the DiaSurg 2 study received a standardized laparoscopic RYGB with a 150 cm alimentary and a 75 cm biliopancreatic limb by the same surgical team. After RYGB, the medical therapy for diabetes, hypertension and other comorbidities was stopped and restarted based on blood glucose values, blood pressure and serum lipids according to an experienced endocrinologist's assessment (PPN, SK). All patients received a multivitamin supplementation (Multibionta, Merck, Darmstadt, Germany) twice daily with regular clinical and laboratory follow-up and additional supplementation as deemed necessary [22].

Patients receiving medical treatment received an insulin-based medical therapy combined with a lifestyle change, targeting HbA1c ≈ 7.5%, based on the available evidence at this time. Additional diabetic therapy such as metformin and others were allowed as deemed necessary by the treating endocrinologist. Treatment of comorbidities such as arterial hypertension or dyslipidemia was also required to achieve the target range recommended by the best available evidence.

### Glucagon Stimulation test

GST was performed after an overnight fast of at least 12 h. Insulin therapy was withheld for at least 12 h prior to testing, and long-acting insulin was omitted 24 h before the test. One milligram of intravenous glucagon was administered, with blood sampling at 0, 1, 5, 10, 15, and 30 min. The test was repeated in all patients after 3, 6, 12 and 24 months in both studies.

### Indices of Insulin Sensitivity and Secretion

HOMA2-indices were calculated using the HOMA2 calculator based on C-peptide [27]. While HOMA1, the original model from Matthews et al. [28], contains a simple mathematical approximation utilizing blood insulin and glucose levels, HOMA2 is an updated computer model of the HOMA1 based on non-linear solutions from the following biomarkers: fasting blood glucose and C-peptide. The HOMA2 Calculator, accessible for free from www.OCDEM.ox.ac.uk (https://www.dtu.ox.ac.uk/homacalculator/download.php), can be utilized for these calculations [27]. However, the absolute value of HOMA indices is dependent on the specific glucose, insulin, and C-peptide assays performed, and there are no universally established thresholds for defining “normal” or “abnormal” levels [29].

The Matsuda-Index (MI) [30, 31] and Disposition-Index (DI) [32, 33] were both calculated using the GST. The respective formulae for calculating MI and DI are provided in the **supplements**. Importantly, these indices were originally developed for oral glucose–based tests (OGTT) and intravenous glucose tolerance tests (IVGTT), and are not validated for intravenous glucagon stimulation. We further acknowledge that glucagon-induced insulin secretion involves distinct physiological pathways compared to glucose-stimulated insulin secretion. The Disposition Index used in this study represents a study-specific adaptation based on GST-derived measures and does not correspond to the original minimal model–based formulation. Accordingly, both indices were used exclusively as exploratory, internally consistent, within-subject descriptors to assess longitudinal changes under identical experimental conditions, and not as validated measures of insulin sensitivity or β-cell function. Remission of T2D was defined according to a new consensus statement from the American Diabetes Association (ADA) and European Association for The Study of Diabetes (EAS) as a return of HbA1c to < 6.5% (< 48 mmol/mol) in the absence of glucose-lowering pharmacotherapy for at least 3 months [34].

Insulin measurements were performed using a standardized clinical immunoassay (Roche Elecsys, Roche Diagnostics), and the same assay was used consistently across all time points.

### Data Analysis

Data are presented as mean ± standard error of the mean (SEM) or as indicated. Given the exploratory nature of the study, the small sample size, and substantial attrition at later follow-up time points, analyses were primarily descriptive.

Formal inferential statistical testing for longitudinal and between-group comparisons was not systematically applied, and results are therefore presented as descriptive trajectories.

For baseline characteristics, group comparisons were performed using appropriate statistical tests (t-test or Mann–Whitney U test for continuous variables and chi-square test for categorical variables) to describe differences at study entry.

GraphPad Prism version 5 (San Diego, USA) and SPSS version 23 (IBM, Armonk, USA) were used for data analysis and visualization. 

## Results

### Baseline Characteristics

Baseline patient characteristics are shown in Table 1. Mean age was 55.5 ± 6.5 years (60% male), BMI 32.4 ± 2.5 kg/m², and HbA1c 8.7 ± 1.3%, with all patients on insulin. Average T2D duration was 12.1 ± 1.1 years and insulin therapy 8.1 ± 1.1 years. Most patients used at least one additional oral glucose-lowering medication, most often metformin. Groups were similar except for age, which was higher in the surgery group. Comorbidities and medication use did not differ.

**Table 1:** Baseline Patients Characteristics for the whole cohort and the Roux-en Y Gastric Bypass Group vs. the Best Medical Care Group

**Baseline characteristics**				
	**All patients (n=35)**	**BMC** ** (n=8)**	**RYGB** ** (n=27) **	**p-value**
Age [y]*	55.5 ± 6.5	50.9 ± 6.5	56.9 ± 5.9	0.017
Sex (m:f) [%]	60:40	75:25	56:44	0.431
BMI [kg/m²]*	32.42 ± 2.5	31.5 ± 2.7	32.7 ± 2.4	0.200
HbA1c [%]*	8.7 ± 1.3	9.2 ± 1.4	8.6 ± 1.2	0.253
OAD°				0.676
- None	17% (6/35)	12.5% (1/8)	18.5% (5/27)	
- One drug	57% (20/35)	50% (4/8)	59% (16/27)	
- Two drugs	26% (9/35)	37.5% (3/8)	22% (6/27)	
- Mean	1.1 ± 0.7	1.3 ± 0.7	1.0 ± 0.6	
Insulin therapy°/*	100% (35/35)	100% (8/8)	100% (27/27)	1.00
- long / IE/d	97% (34/35) / 41.6 ± 34.6	100% (8/8) / 35.3 ± 29.1	96% (26/27) / 43.7 ± 36.7	1.00 / 0.492
- short / IE/d	69% (24/35) / 24.5 ± 28.5	75% (6/8) / 24.8 ± 19.4	67% (18/27) / 24.3 ± 31.5	1.00 / 0.629
Co-morbidities°				
aHT	89% (31/35)	88% (7/8)	89% (24/27)	1.000
CHD	6% (2/35)	0	7% (2/27)	1.000
CVI	17% (6/35)	0	22% (6/27)	0.299
Dyslipidemia	40% (14/35)	38% (3/8)	41% (11/27)	1.000
OSAS	6% (2/35)	13% (1/8)	4% (1/27)	0.410
Number of medication*				
aHT	1.9 ± 1.3	1.4 ± 1.1	2.1 ± 1.2	0.191
CHD/CVI	0.4 ± 0.5	0.3 ± 0.5	0.5 ± 0.5	0.390
Dyslipidemia	0.4 ± 0.5	0.5 ± 0.5	0.4 ± 0.5	0.765

*mean ±standard deviation, ° % of patients (n°/total), *BMC* Best Medical Care, RYGB: Roux-en Y Gastric Bypass, *aHT* arterial hypertension, *CHD* coronary heart disease, *CVI* cerebrovascular insufficiency, *OAD* oral antidiabetic medication, *OSAS* obstructive sleep apnea

For the results presented below, we note that substantial attrition in the BMC group at later follow-up points, especially at 12 and 24 months, limits the robustness of the data. Accordingly, these findings should be interpreted descriptively.

### Glycemic Outcomes and Medication Use

**Fig. 1:**
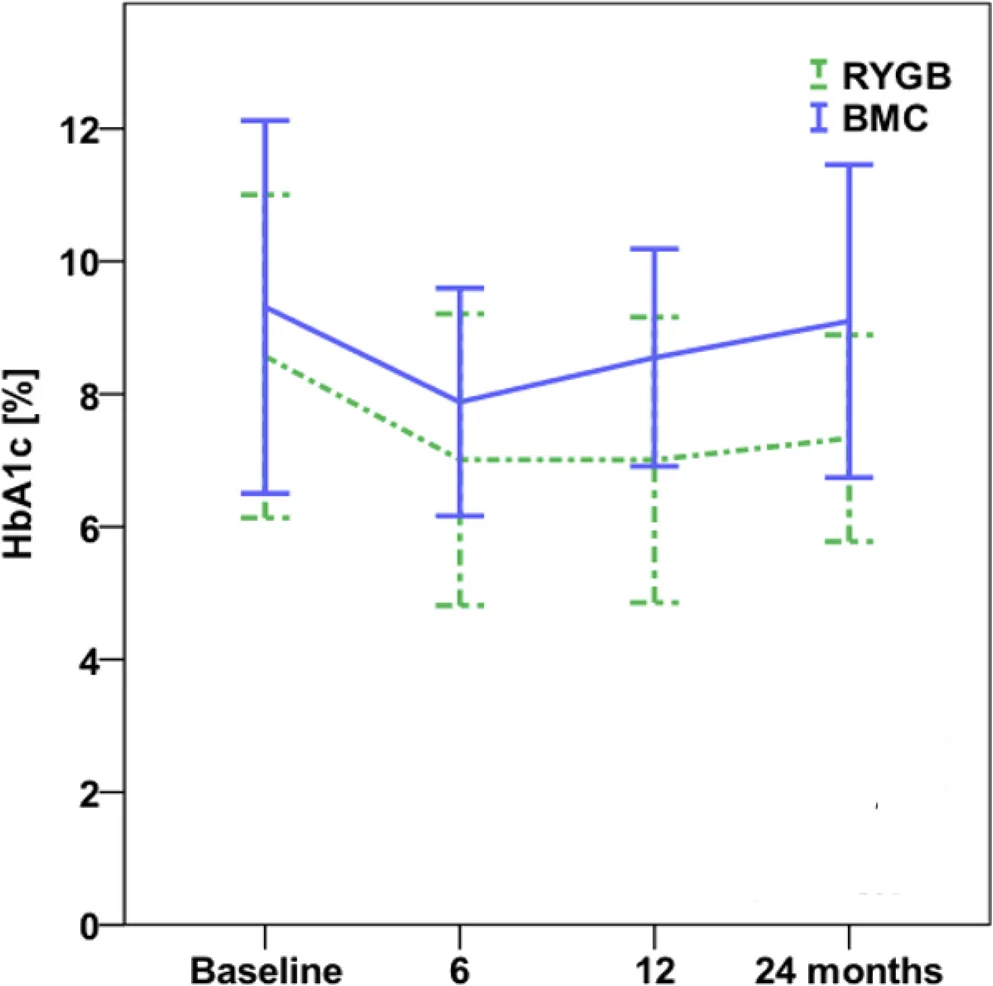
HbA1c Comparing Patients after Best Medical Care (BMC) vs. Roux-en Y Gastric Bypass (RYGB). Data are shown as mean ± 2x standard error of the mean

Changes in HbA1c, T2D remission, weight loss and medication are summarized in Table 2 and should be interpreted descriptively due to differences in study design and follow-up. In the conservative group, no patient achieved diabetes remission and HbA1c did not change substantially after 12 and 24 months. Insulin therapy was stopped in one-third of patients that completed the 24 months follow-up in the conservative group while all of them required at least one oral antidiabetic drug. BMI decrease was only clinically relevant in the RYGB group. Likewise, RYGB was associated with higher Total Weight Loss (%TWL) and Excess Weight Loss (%EWL) at all follow-up points. Medication used to control cardiovascular comorbidities remained unchanged. In the surgery group, 10% of patients had a T2D-remission after 24 months while the HbA1c decreased from 8.6 ± 1.2% to 7.3 ± 0.8% (Fig. 1). After 24 months, 73% of patients no longer required insulin treatment which was stopped at a HbA1c of 7.5%. In addition, a third of patients did not require additional oral medication. In total, 23% of patients after RYGB were off any antidiabetic medication. The BMI also decreased after RYGB from 32.7 ± 2.4 kg/m^2^ to 24.0 ± 2.5 kg/m^2^ after 24 months. Medication used to control cardiovascular comorbidities also decreased from an average of 3.0 ± 1.7 medications to 1.8 ± 1.8.

**Table 2:** Changes in Glycemic Control, Body Mass Index, Weight Loss and Medication over Time After Roux-en Y Gastric Bypass vs. Best Medical

Antidiabetic medication over time										
	BMC (Best Medical Care)					RYGB (Roux-en Y Gastric Bypass)				
	Baseline n=8	3 months n=2	6 months n=5	12 months n=4	24 months n=3	Baseline n=27	3 months n=23	6 months n=26	12 months n=25	24 months n=22
OAD*	1.3±0.7	1±0	1.4±0.9	1.8±1.0	2±1	1.0±0.6	0.4±0.5	0.6±0.6	0.6±0.6	0.7±0.6
None°	12%(1/8)	0	0	0	0	18%(5/27	57%(13/23	46%(12/26)	48%(12/25)	32%(7/22)
One°	50%(4/8)	100%(2/2)	80%(4/5)	50%(2/4)	33%(1/3)	59%(16/27)	43%(10/23)	46%(12/26)	48%(12/25)	64%(14/22)
Two°	38%(3/8)	0	0	25%(1/4)	33%(1/3)	22%(6/27)	0	8% 2/26)	4%(1/25)	4%(1/22)
Three°	0	0	20%(1/5)	25%(1/4)	33%(1/3)	0	0	0	0	0
Insulin°	100%(8/8)	100%(2/2)	60%(3/5)	75%(3/4)	67%(2/3)	100%(27/27)	43%(10/23)	19%(5/26)	20%(5/25)	27%(6/22)
HbA1c [%]*	9.3±-1.4	8.4±1.6	7.9±0.9	8.6±0.8	9.1±1.2	8.6%±1.2	7.5%±1.3	7.0%±1.1	7.0%±1.1	7.3%±0.8
Diabetes remission°		0%(0/2)	0%(0/5)	0%(0/4)	0%(0/3)		13% (3/23)	27% (7/26)	28% (7/25)	10% (2/20)
BMI*	31.5 ± 2.7	30.7 ± 3.0	30.6 ± 3.1	30.6 ± 3.1	30.6 ± 3.1	32.7 ± 2.4	27.6 ± 2.7	25.9 ± 2.4	24.7 ± 1.8	24.0 ± 2.5
%TWL	-	2.4 ± 4.2	2.5 ± 4.2	2.6 ± 4.2	2.7 ± 4.4	-	15.4 ± 5.7	20.8 ± 8.7	23.7 ± 6.4	25.0 ± 9.4
%EWL	-	15.4 ± 21.7	16.0 ± 22.7	16.7 ± 23.8	18.0 ± 26.3	-	70.3 ± 28.3	89.4 ± 41.0	107.7 ± 27.6	113.9 ± 32.6
Other Medication	2.1 ± 1.5	2.3 ± 3.3	0.5 ± 0.7	3 ± 3.6	-	3 ± 1.7	1.2 ±1.3	1.5 ±1.5	1.3 ±1.5	1.8 ± 1.8

*mean ±standard deviation, ° % of patients (n°/total, Diabetes remission HbA1c <6.5% without need of medication, Other medication Medication for control of cardiovascular risk factors (e.g. for *aHT*, *CHD*, *CVI* and *DLP*), *OAD* oral antidiabetic medication, *aHT* arterial hypertension, *CHD* coronary heart disease, *CVI* cerebrovascular insufficiency, *DLP* dyslipidemia, *%EWL* Percentage Excess Weight Loss , *%TWL* Percentage Total Weight Loss

Mean HbA1c was comparable between RYGB and BMC at baseline while RYGB was associated with lower HbA1c and reduced use of oral anti-diabetic medication over the 24-month follow-up compared with BMC (Fig. 1; Table 2). Additionally, a trend toward reduced insulin use and number of medications for control of cardiovascular risk factors or T2D remission was observed.

Table 3 provides an overview of the C-peptide based HOMA-indices before therapy and during the follow-up. HOMA2-derived indices showed differing trajectories between treatment groups.

**Table 3:** Changes in HOMA2-Indices After Roux-en Y Gastric Bypass vs. Best Medical Care

HOMA-2										
	BMC (Best medical care)					RYGB (Roux-en Y Gastric Bypass)				
	Baseline *n* = 8	3 months *n* = 2	6 months *n* = 5	12 months *n* = 4	24 months *n* = 2	Baseline *n* = 24	3 months *n* = 21	6 months *n* = 19	12 months *n* = 19	24 months *n* = 24
HOMA-2-B	68.1 ± 23.5	50.3 ± 12.2	132.6 ± 57.6	66.7 ± 54.1	90.9 ± 89.2	97.3 ± 56.4	130.3 ± 69.6	128.9 ± 63.2	134.3 ± 71.0	126.3 ± 52.2
HOMA-2-S	29.6 ± 52.7	24.9 ± 5.9	16.1 ± 8.9	28.9 ± 17.7	16.7 ± 6.9	21.9 ± 18.9	20.3 ± 9.7	25.5 ± 12.1	30.7 ± 27.9	31.0 ± 29.1
HOMA-2-IR	11.6 ± 10.7	4.1 ± 1.0	7.3 ± 2.7	4.7 ± 3.3	6.6 ± 2.7	7.2 ± 5.3	5.9 ± 2.6	4.6 ± 1.8	4.2 ± 1.5	4.4 ± 1.8

* mean ±standard deviation, ° % (n/total)

In the RYGB group, HOMA2-B increased within the first months after surgery and remained elevated over 24 months, while HOMA2-IR decreased and remained lower compared to baseline. HOMA2-S showed a gradual increase over time, indicating improved insulin sensitivity.

In contrast, the BMC group exhibited variable and non-uniform changes across all HOMA2 indices. HOMA2-B showed a transient increase at 6 months followed by fluctuating values at later time points, while HOMA2-S and HOMA2-IR varied without a consistent directional pattern.

### Glucagon Stimulation Test

**Fig. 2:**
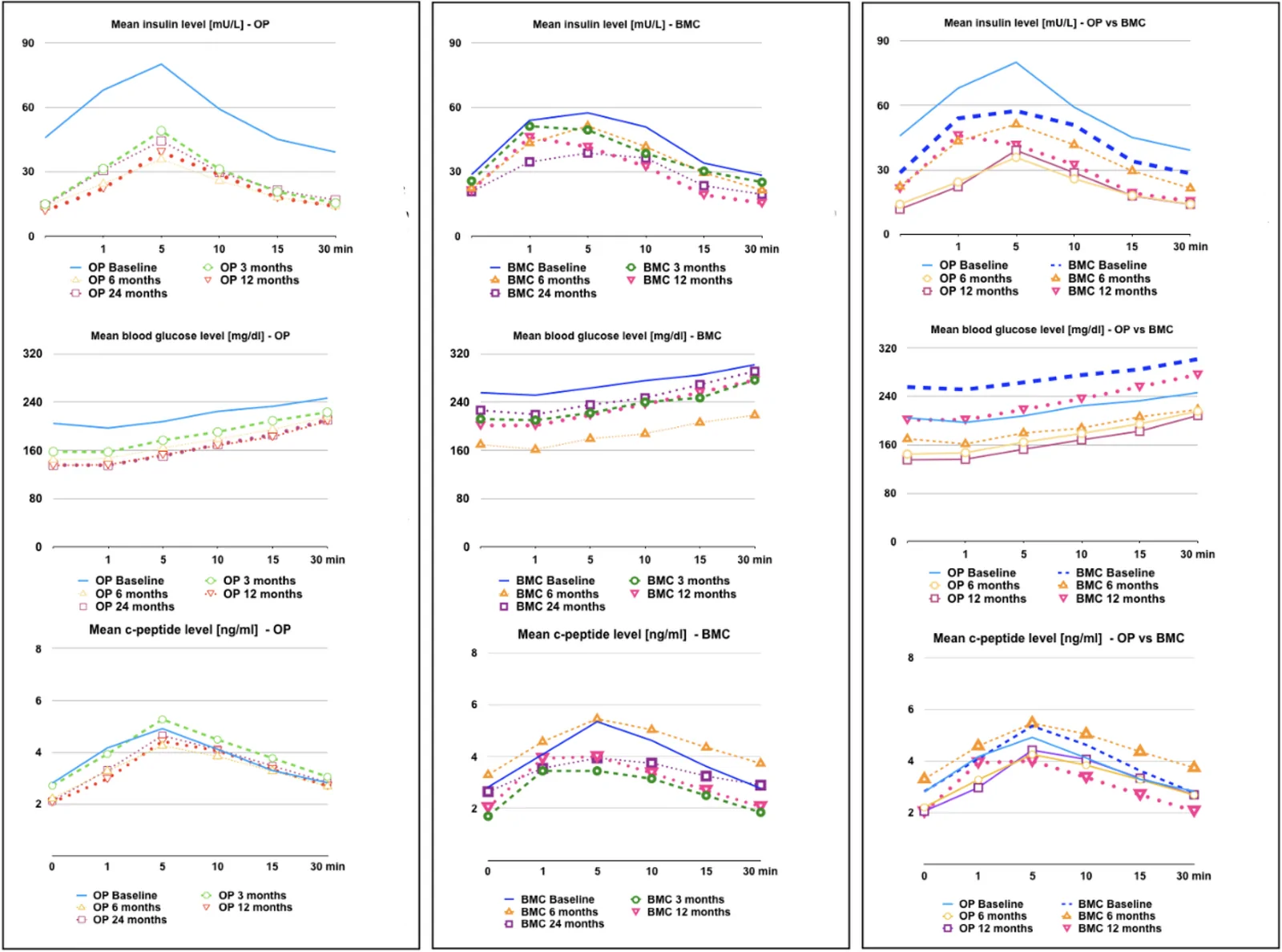
Glucagon Stimulation Test Comparing Patients after Best Medical Care vs. Roux-Y Gastric Bypass. 30-min glucagon stimulation test in the Roux-en Y gastric bypass (RYGB) and best medical care (BMC) groups are shown for insulin, glucose, and C-Peptide. Comparisons of baseline, 6, and 12 months are shown.

Changes in the GST are shown in Fig. 2. RYGB was associated with a rapid reduction in insulin secretion within 3 months. The AUC for insulin secretion during the 30-min glucagon test remained at a lower level over 24 months while the C-peptide levels did not show a substantial change. Similarly, the AUC of glucose as a marker for glycemic control [35], decreased rapidly within 3 months and remained at lower levels after 24 months. BMC was also associated with a reduction in insulin AUC. Similar to the RYGB group, no substantial changes in C-peptide AUC were observed over the study period. In contrast to RYGB, glucose AUC in the BMC group showed a meaningful decrease only after 6 months and subsequently returned to levels comparable to baseline.

**Fig. 3:**
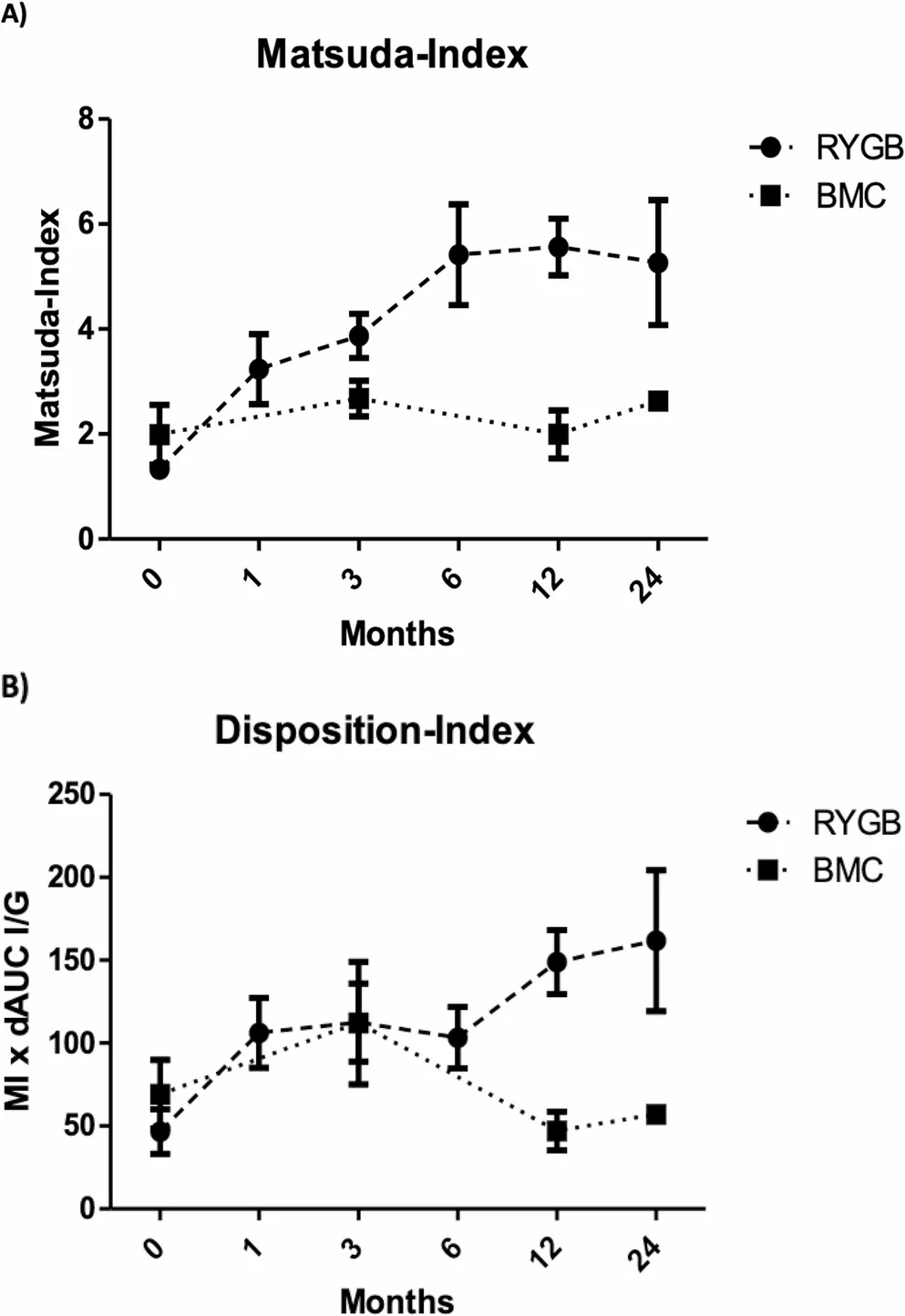
Matsuda (**A**)- and Disposition Index (**B**) after RYGB vs. BMC. Matsuda-Index (A) and Disposition-Index (B) based on the Glucagon Stimulation Test in the Roux-en Y gastric bypass (RYGB) and best medical care (BMC) groups are shown

Using the GST-based MI and DI as exploratory, non-validated indices, RYGB was associated with an increase in MI from 1.33 ± 0.19 to 5.27 ± 1.19 after 24 months (Fig. 3). The DI was also improved by RYGB from 46.66 ± 13.43 before RYGB to 161.8 ± 42.56 after 24 months. BMC did not have a relevant impact on insulin resistance based on the MI. MI was 1.99 ± 0.59 before study inclusion and changed to 2.00 ± 0.46 after 12 months while DI decreased from baseline (69.15 ± 20.65) to 41.96 ± 18.18 after 12 months. There was no difference between RYGB vs. BMC at baseline regarding MI and DI but RYGB showed numerically larger increases in MI and DI compared to BMC.

### Subgroup Analyses of Preoperative Pancreatic Function

When stratified by preoperative pancreatic function, patients with poor function showed no sustained T2D remission but experienced substantial improvements in glycemic control, insulin discontinuation, BMI, and recovery of C-peptide–based indices after RYGB, approaching values similar to those observed in patients with good pancreatic function at 24 months. Analyses stratified by preoperative pancreatic function and exploratory indices were underpowered and are therefore presented for descriptive and hypothesis-generating purposes only and are displayed in detail in the Supplementary Material.

## Discussion

This study provides a descriptive and exploratory analysis of longitudinal changes in glycemic control and insulin secretion dynamics following RYGB compared with best medical care in non-severely obese, insulin-treated patients with T2D. Using an intravenous GST with follow-up extending to 24 months, we aimed to characterize physiological response patterns under standardized conditions rather than to establish causal or mechanistic effects. All physiological observations should be interpreted as descriptive and exploratory, and between-group comparisons are observational rather than randomized.

The patient cohorts analyzed in this study (DiaSurg 1 and DiaSurg 2) have been reported in several prior publications focusing on clinical outcomes, including glycemic control, neuropathy, renal function, nutritional status, and liver disease [21–26]. The present analysis represents a secondary, hypothesis-generating evaluation of these cohorts with a distinct physiological focus. In contrast to these previous reports, which primarily assessed clinical and fasting metabolic parameters, this study specifically investigates longitudinal changes in insulin secretion dynamics and glucose responses under standardized intravenous stimulation using GST, with repeated measurements up to 24 months. To our knowledge, such long-term intravenous physiological data have not previously been reported in this population. Most prior studies have relied on OGTT or mixed-meal tests, which makes it difficult to isolate RYGB’s direct impact on pancreatic function. Our approach may help overcome this limitation.

The principal physiological observation of this study was a rapid and sustained reduction in insulin secretion after RYGB, as reflected by a marked decrease in insulin AUC during GST, accompanied by improved glycemic control. Importantly, this reduction in insulin secretion occurred without a corresponding sustained increase in C-peptide responses. This pattern is consistent with reduced insulin demand and improved insulin efficiency. While modest increases in C-peptide were observed in some patients, these findings should not be interpreted as definitive evidence of β-cell recovery or regeneration. Accordingly, our findings suggest that improved glycemic control after RYGB in this group of patients may be primarily driven by enhanced insulin sensitivity and reduced metabolic burden on the β-cell. An alternative explanation for the observed divergence between insulin and C-peptide responses is altered hepatic insulin clearance [36]. As insulin undergoes substantial first-pass hepatic extraction whereas C-peptide does not, changes in hepatic insulin clearance after RYGB may contribute to lower circulating insulin levels independent of changes in insulin secretion [37]. Since the GST does not allow differentiation between insulin secretion and clearance, this mechanism cannot be excluded.

However, although glycemic control improved overall following RYGB, the proportion of patients achieving formal diabetes remission declined between 12 and 24 months. This apparent discrepancy reflects the important distinction between sustained metabolic improvement and remission as defined by strict clinical criteria. Consistent with previous evidence, improvements in glucose metabolism after MBS are largely driven by enhanced β-cell function and insulin sensitivity, yet remission remains strongly influenced by baseline disease severity and progression [38]. The observed decline in remission rates over time may therefore be attributable to the progressive nature of long-standing type 2 diabetes, including ongoing β-cell dysfunction, as well as the need to reintroduce glucose-lowering therapies in some patients. These findings suggest that, while RYGB can induce durable improvements in glycemic control, such metabolic benefits do not necessarily translate into sustained remission for all individuals, particularly those with more advanced disease.

Our findings should also be interpreted in the context of recent studies using alternative methodological approaches. Studies employing gold-standard clamp techniques have demonstrated rapid improvements in β-cell function and insulin sensitivity after MBS [39], while others suggest that weight loss per se may account for a substantial proportion of metabolic improvements [40, 41]. Bradley et al. demonstrated that improvements in insulin sensitivity are largely proportional to the degree of weight loss and are comparable across procedures when matched for weight reduction, suggesting that weight loss per se is a primary driver of enhanced insulin action [41].

In contrast, Prasad et al. reported that diabetes remission after RYGB is primarily determined by improvements in β-cell function, whereas changes in body weight and insulin sensitivity did not differ between remission groups and were not predictive of outcome [42]. Taken together, these findings support a model in which weight loss predominantly governs improvements in insulin sensitivity, while recovery of β-cell function represents the key determinant of glycemic outcomes. Our results are consistent with this framework, suggesting that inter-individual differences in β-cell functional capacity may underlie the observed heterogeneity in metabolic response. This distinction is particularly relevant for interpreting our stratification based on β-cell function.

In addition, Haraguchi et al. [43] demonstrated that glucagon-based stimulation may underestimate insulin secretory capacity under conditions of increased incretin signaling. In this context, the present GST-based findings should be interpreted as complementary physiological observations under intravenous conditions rather than as direct measures of β-cell function or insulin sensitivity comparable to clamp-based methodologies.

Exploratory indices derived from the GST, including the Matsuda Index and Disposition Index, increased after RYGB but not after BMC. These indices were originally developed and validated for oral glucose–based tests and/or intravenous glucose tolerance test (IVGTT) (DI) [30–33] and are not established for glucagon stimulation. Given the lack of validated reference ranges or clinically meaningful thresholds for GST-derived indices, the observed changes in MI and DI cannot be interpreted in terms of clinical significance and should be viewed as descriptive markers of longitudinal change only rather than definitive measures of whole-body insulin sensitivity or β-cell function. When applied consistently within subjects over time using the same stimulation protocol and assays, however, these indices provide internally consistent information on trends over time. In this context, the observed increases after RYGB support the interpretation of reduced insulin requirements in the setting of improved glycemic control.

In contrast, patients treated with BMC showed less consistent physiological changes. Although modest reductions in insulin AUC were observed at later follow-up time points, these were not accompanied by sustained improvements in glucose AUC or by consistent changes in exploratory indices. While these divergent response patterns suggest greater durability of glycemic improvement after RYGB, between-group comparisons must be interpreted cautiously, as they are observational and non-randomized.

Subgroup analyses were performed to explore whether baseline pancreatic function influenced postoperative responses to RYGB. Patients with preserved preoperative pancreatic function exhibited an early and pronounced reduction in insulin secretion after surgery, whereas patients with lower preoperative pancreatic function demonstrated a more gradual decline in insulin AUC, accompanied by modest increases in C-peptide responses over time. Despite differing hormonal trajectories, improvements in glycemic control were broadly similar across subgroups, although patients with lower preoperative pancreatic function were more likely to require continued insulin therapy.

These findings suggest that RYGB may improve glycemic control across different physiological phenotypes of T2D [44]. However, these subgroup analyses are underpowered and should be considered hypothesis-generating only. Additional exploratory analyses stratified by baseline Disposition Index showed larger relative increases in patients with lower baseline values and smaller changes in those with higher baseline values. Given the small sample sizes, attrition at later time points, and lack of validation of GST-derived indices, these observations should be interpreted cautiously and not as evidence of differential treatment effects.

Taken together, our results extend previous short-term physiological studies using intravenous or clamp-based approaches [17–19] by providing longer-term descriptive data under standardized, intravenous conditions in a clinically relevant but understudied population of non-severely obese, insulin-treated patients with T2D. The findings emphasize reduced insulin demand and changes consistent with improved metabolic efficiency as key features of sustained glycemic improvement after RYGB, while highlighting the need for larger, methodologically controlled studies to further elucidate underlying mechanisms.

### Limitations

This study has several important limitations that should be considered when interpreting the findings.

First, the comparison between RYGB and BMC does not represent a fully randomized, balanced design. The surgical group includes patients from both DiaSurg 1 (a single-arm cohort) and DiaSurg 2 (a randomized trial), whereas the BMC group originates exclusively from the randomized trial and exhibited higher attrition and shorter follow-up. The combination of patients from these two studies introduces potential heterogeneity in patient selection, study design, and clinical management. This pooling approach may introduce bias and further limits comparability between groups. Although pooling DiaSurg 1 and DiaSurg 2 increased sample size, the comparability of the two cohorts requires careful consideration. Both studies enrolled patients with non-severe obesity and long-standing T2D treated with insulin and applied the same surgical procedure and standardized follow-up protocols, supporting a broadly comparable clinical phenotype. Baseline metabolic characteristics, including BMI, HbA1c, and insulin treatment, were similar between groups, although patients in DiaSurg 2 were required to have microvascular complications and the pooled RYGB group was older on average. These differences may reflect partially distinct patient populations. Therefore, while pooling can be justified for exploratory physiological analyses under similar experimental conditions, it remains a pragmatic approach and may introduce bias.

Additionally, baseline differences between groups, most notably a higher mean age in the RYGB group, may have influenced metabolic outcomes, as age affects insulin sensitivity and β-cell function. Consequently, all between-group comparisons are observational in nature and do not support causal inference.

Second, the sample size was limited, particularly at later follow-up time points, with substantial attrition by 24 months. The high attrition rate in the BMC group at these later stages further reduces the validity of between-group comparisons and necessitates a primarily descriptive interpretation of the data. Accordingly, all subgroup findings should be interpreted as exploratory.

Third, the Matsuda Index and Disposition Index used in this study were derived from GST data and are not validated for this testing modality. The applied DI represents a study-specific adaptation and does not correspond to the original minimal model–based formulation. Furthermore, due to the distinct physiological mechanisms of glucagon- versus glucose-stimulated insulin secretion, these indices cannot be interpreted within established OGTT- or IVGTT-based frameworks. While their consistent application within subjects over time allows descriptive assessment of longitudinal change, these indices should not be interpreted as validated measures of insulin sensitivity or β-cell function, nor should established cut-offs derived from oral glucose–based studies be applied.

Fourth, while GST avoids acute enteral stimulation, it does not eliminate chronic postoperative influences of gut hormones, bile acids, or neural mechanisms on glucose metabolism. Therefore, mechanistic conclusions—particularly regarding GLP-1 independence—cannot be drawn from this study.

Fifth, interpretation of glucagon-stimulated insulin secretion is further complicated by altered incretin physiology following RYGB. Glucagon may stimulate insulin secretion partly via GLP-1 receptor–dependent mechanisms, and endogenous GLP-1 levels are markedly increased after RYGB. Emerging evidence suggests that GST may underestimate or otherwise alter the assessment of insulin secretory capacity in the presence of enhanced incretin signaling [43]. As a result, GST responses in the postoperative setting may reflect not only intrinsic β-cell responsiveness but also changes in incretin receptor activity and the broader hormonal milieu, thereby limiting mechanistic interpretation.

Sixth, the study population consisted of patients with non-severe obesity and insulin-treated long-standing T2D, which may limit generalizability to individuals with earlier disease stages or higher degrees of obesity. In addition, the requirement for preserved insulin secretion (glucagon-stimulated C-peptide > 1.5 ng/ml) may have introduced selection bias by excluding patients with advanced β-cell failure, further restricting generalizability to the broader population of patients treated with insulin.

Additionally, potential cross-reactivity of the insulin assay with exogenous insulin analogues and residual circulating insulin cannot be fully excluded, particularly at baseline. As insulin undergoes first-pass hepatic extraction and assay characteristics may vary, measured insulin concentrations may be influenced by both biological and analytical factors. However, insulin therapy was withheld prior to testing, and the consistent use of the same assay across all time points supports the validity of longitudinal within-subject comparisons.

Finally, the medical therapy applied reflects the standard of care at the time of study initiation and does not include more recent glucose-lowering agents such as GLP-1 receptor agonists or SGLT2 inhibitors. Therefore, comparisons between RYGB and medical therapy may not fully reflect current treatment paradigms.

## Conclusion

In this descriptive, exploratory analysis, RYGB was associated with sustained improvements in glycemic control and reduced insulin secretion requirements in patients with non-severe obesity and insulin-treated T2D. Under intravenous stimulation testing conditions, insulin demand decreased while C-peptide responses were largely preserved, changes that are consistent with improved metabolic efficiency. Overall, these findings should be interpreted as hypothesis-generating and require confirmation in adequately powered, randomized studies using validated measures of insulin sensitivity and β-cell function.

## Supplementary Information

Below is the link to the electronic supplementary material.

Supplementary Material 1 (DOCX 2.92 MB)

## Data Availability

The data and codes relating to the study are available from the corresponding author upon reasonable request.

## Declarations

### Data Access and Responsibility

Adrian T. Billeter is the guarantor of this work and, as such, had full access to all the data in the study and takes responsibility for the integrity of the data and the accuracy of the data analysis.

### Data Resource Sharing and Availability

The data and codes relating to the study are available from the corresponding author upon reasonable request.

## **Abbreviations**

ADA: American Diabetes Association
AUC: Area Under the Curve
BMC: Best Medical Care / Best Medical Therapy
BMI: Body Mass Index
DI: Disposition Index
DFG: Deutsche Forschungsgemeinschaft (German Research Society)
EAS: European Association for the Study of Diabetes
EWL: Excess Weight Loss
GLP-1: Glucagon-Like Peptide-1
GST: Glucagon Stimulation Test
HbA1c: Glycated Hemoglobin
HOMA: Homeostasis Model Assessment
HOMA1: Original Homeostasis Model Assessment (Matthews et al.)
HOMA2: Updated Computer Model of HOMA
HOMA2-B: HOMA2 β-cell Function Index
HOMA2-IR: HOMA2 Insulin Resistance Index
IR: Insulin Resistance
IVGTT: Intravenous glucose tolerance test
MI: Matsuda Index
OGTT: Oral Glucose Tolerance Test
PF: Pancreatic Function
RCT: Randomized Controlled Trial
RYGB: Roux-en-Y Gastric Bypass
SD: Standard Deviation
SEM: Standard Error of the Mean
SIDD: Severe Insulin-Deficient Diabetes
SIRD: Severe Insulin-Resistant Diabetes
SPSS: Statistical Package for the Social Sciences
T2D: Type 2 Diabetes
TWL: Total Weight Loss
