# Supplementary material for: Effects of Gastric Bypass on Beta-cell function and Insulin sensitivity in Patients with Non-Severe Obesity (BMI<35 kg/m2) and Insulin-treated Type 2 Diabetes
Source: Obes Surg. 2026 Apr 27;36(6):3015–26. doi: 10.1007/s11695-026-08702-5 (PMC13653954; doi:10.1007/s11695-026-08702-5)
Supplement: Supplementary file 1 [file 11695_2026_8702_MOESM1_ESM.docx]

Table of Contents

[Supplementary Figure S1: Formula for calculation of Matsuda-Index	2](#_Toc227145542)

[Supplementary Figure S2: Formula for calculation of Disposition-Index	2](#_Toc227145543)

[Subgroup analyses of preoperative pancreatic function	3](#_Toc227145544)

[Supplementary Figure S3: HbA1c Comparing patients with bad vs. good preoperative pancreatic function after Roux-en Y Gastric Bypass	6](#_Toc227145545)

[Supplementary Figure S4: Glucagon stimulation test in patients with bad vs. good preoperative pancreatic function after Roux-en Y Gastric Bypass	7](#_Toc227145546)

[Supplementary Table S1: Changes in Glycemic Control, Body Mass Index, and Medication over Time depending on preoperative pancreatic function	8](#_Toc227145547)

# Supplementary Figure S1: Formula for calculation of Matsuda-Index

Note: The original Matsuda Index was developed for OGTT-derived variables. In this study, analogous mean insulin and glucose values derived from the glucagon stimulation test (GST) were used for exploratory purposes.

# Supplementary Figure S2: Formula for calculation of Disposition-Index

Note: The original Disposition Index was developed using IVGTT-based minimal model analysis. The formulation used here represents a study-specific, non-validated adaptation based on GST-derived variables and is applied for exploratory, within-subject comparisons only.

# Subgroup analyses of preoperative pancreatic function

Analyses stratified by preoperative pancreatic function and exploratory indices were underpowered and are presented for descriptive and hypothesis-generating purposes only.

Preoperative pancreatic function (PF) was categorized by 5min stimulated C-peptide during the GST. Preoperative pancreatic function (PF) was categorized by 5min stimulated C-peptide during the GST. A cutoff of 2.9 ng/ml was selected pragmatically to stratify patients into groups with relatively preserved (good) versus reduced (poor) β-cell function. This value corresponds approximately to 1.0 nmol/L and lies well above ranges associated with insulin deficiency (e.g., <0.6 nmol/L in stimulated conditions), which have been reported to indicate preserved endogenous insulin secretion [1].

This range is also consistent with values used in clinical practice to identify patients with meaningful residual β-cell function. Importantly, the study population was restricted to individuals with preserved insulin secretion, defined by a glucagon-stimulated C-peptide >1.5 ng/ml as an inclusion criterion. Within this non-deficient population, the cutoff of 2.9 ng/ml was applied to further stratify patients into groups with relatively lower versus higher β-cell functional capacity. However, absolute C-peptide thresholds depend on assay characteristics, stimulation method, and study population, and significant inter-assay variability persists due to the lack of standardization of C-peptide measurements. Consequently, published cutoffs are not directly transferable between settings and should be interpreted with caution. As no validated cutoff exists for GST-based stratification using absolute stimulated C-peptide values, the threshold applied in this study should be considered exploratory, internally defined, and intended for internal comparison rather than clinical decision-making.

Eighteen patients had a good and five patients a poor PF. The changes in HbA1c, T2D remission, and antidiabetic medication over time is provided in Supp. **Table S1.** In the poor PF group, no patient achieved diabetes remission after 24 months while one out of the five patients had a short term T2D remission between three to twelve months. HbA1c changed significantly after 6 and 12 months, respectively. After 24 months the change was barely not significant. However, 60% of patients were off insulin therapy over the 24 months follow-up in the bad PF group while 80% required one oral antidiabetic drug and one patient was completely off any antidiabetic medication with a HbA1c of 6.5%. BMI dropped from 33.3 ± 2.6 kg/m^2^ to 25.7 ± 2.5 kg/m2 while there were no changes in medications for control of cardiovascular risk factors. In the good PF group, 14% achieved remission, 80% discontinued insulin, and BMI decreased from 32.5 to 24.1 kg/m². Five of the fifteen patients were of any oral antidiabetic medication while the remaining patients required up to two different anti-diabetic medications. The BMI also dropped significantly after RGYB from 32.5 ± 2.3 kg/m2 to 24.1 ± 2.5 kg/m^2^.

Mean HbA1c was comparable between patients with bad vs. good PF at baseline as well as during the follow-up **(Supp. Figure S3)**. There was no difference in number of medications, insulin use or T2D-remission.

Changes in the GST after RYGB based on preoperative PF are shown in **Supp.** **Figure S4**. Patients with good PF showed a significant decrease in the insulin AUC after 3 months that stayed stable over 24 months. Patients with bad PF showed a barely non-significant decrease in insulin AUC after 3 months, the values after 6 and 12 months were significantly lower. At baseline, the insulin AUC was non-significantly higher in patients with bad PF compared to such with good function. The C-peptide AUC in patients with good PF showed a non-significant increase after 3 months which turned to lower AUCs after 6, 12 and 24 months. In contrast, patients with bad PF showed a significantly lower C-peptide AUC at baseline compared to such with good PF. However, C-peptide AUC increased to comparable values to patients with good PF over the follow-up time. The glucose AUC showed a significant decrease over the follow-up time in patients with good PF. Interestingly, it did not change significantly over time in patients with bad PF.

Low preoperative DI patients showed a marked increase in DI after 24 months from 16.99 ± 2.40 to 196.06 ± 90.72 after 24 months, while patients with a good DI before RYGB had no significant change (67.41 ± 20.66 to 108.6 ± 31.49). Baseline differences between groups disappeared after 24 months. MI did not differ by PF at any time.

**References**

1.	Schleicher E, Kabytaev K, Nauck M, Müller-Wieland D, Peter A, Little RR, Heinemann L (2025) Call for Standardization of C-Peptide Measurement. J Diabetes Sci Technol 19322968251362848. https://doi.org/10.1177/19322968251362848

# Supplementary Figure S3: HbA1c Comparing patients with bad vs. good preoperative pancreatic function after Roux-en Y Gastric Bypass

Data are shown as mean ± 2x standard error of the mean.

# Supplementary Figure S4: Glucagon stimulation test in patients with bad vs. good preoperative pancreatic function after Roux-en Y Gastric Bypass

30min glucagon stimulation test in patients with bad (BPF) vs. good (GPF) preoperative pancreatic function after Roux-en Y gastric bypass (RYGB) are shown for insulin, glucose, and C-Peptid. Comparisons of baseline, 6, and 12 months are shown.

# Supplementary Table S1: Changes in Glycemic Control, Body Mass Index, and Medication over Time depending on preoperative pancreatic function

| **Antidiabetic medication over time** | | | | | | | | | | | |
| --- | --- | --- | --- | --- | --- | --- | --- | --- | --- | --- | --- |
|  | **Bad pancreatic function** | | | | |  | **Good pancreatic function** | | | | |
|  | Baseline  n=5 | 3 months  n=4 | 6 months  n=5 | 12 months  n=5 | 24 months  n=5 |  | Baseline  n= 18 | 3 months  n=16 | 6 months  n=18 | 12 months  n=17 | 24 months  n=15 |
| **OAD***  **None°**  **One°**  **Two°** | 1 ± 0.7  20% (1/5)  60% (3/5)  20% (1/5) | 0.5 ± 0.6  50% (2/4)  50% (2/4)  0 | 0.4 ± 0.5  60% (3/5)  40% (2/5)  0 | 0.6 ± 0.5  40% (2/6)  60% (3/6)  0 | 0.8 ± 0.5  20% (1/5)  80% (4/5)  0 |  | 1 ± 0.6  22% (4/18)  61% (11/18)  17% (3/18) | 0.4 ± 0.5  56% (9/16)  44% (7/16)  0 | 0.6 ± 0.6  44% (8/18)  50% (9/18)  6% (1/18) | 0.5 ± 0.6  53% (9/17)  41% (7/17)  6% (1/17) | 0.7 ± 0.6  33% (5/15)  60% (9/15)  7% (1/15) |
| **Insulin°** | 100% (5/5) | 50% (2/4) | 40% (2/5) | 40% (2/5) | 40% (2/5) |  | 100%(18/18) | 38% (6/16) | 11% (2/18) | 12% (2/17) | 20% (3/15) |
| **HbA1c [%]*** | 8.4±1.4 | 7.4±1.5 | 7.2±1.2 | 7.6±1.5 | 7.8±1.1 |  | 8.9±1.3 | 7.4±1.1 | 6.9±1 | 6.8±0.9 | 7.2±0.7 |
| **Diabetes remission°** |  | 25% (1/4) | 20% (1/4) | 20% (1/5) | 0% (0/4) |  |  | 13% (2/16) | 29% (5/17) | 35% (6/17) | 14% (2/14) |
| **BMI*** | 33.3 ± 2.6 | 26.3 ± 2.8 | 26.5 ± 2.4 | 26.2 ± 1.5 | 25.7 ± 2.5 |  | 32.5 ± 2.3 | 27.5 ± 1.8 | 25.9 ± 2.3 | 24.4 ± 1.7 | 24.1 ± 2.5 |
| **Other Medication*** | 3 ± 1.8 | 0.5 ± 0.5 | 1.4 ± 1.7 | 1.6 ± 2.5 | 4 |  | 2.9 ± 1.6 | 1.4 ± 1.3 | 1.1 ± 1.3 | 1.2 ± 1.3 | 1.6 ± 1.8 |
| - mean ±standard deviation   ° % (n/total)  Diabetes remission: HbA1c <6.5% without need of medication  Other medication: Medication for control of cardiovascular risk factors (e.g. for aHT, CHD, CVI and DLP) | | | | | | | OAD oral antidiabetic medication  aHT arterial hypertension  CHD coronary heart disease  CVI cerebrovascular insufficiency  DLP dyslipidemia | | | | |
